# Beyond bigrams: call sequencing in the common marmoset (*Callithrix jacchus*) vocal system

**DOI:** 10.1098/rsos.240218

**Published:** 2024-11-06

**Authors:** Alexandra B. Bosshard, Judith M. Burkart, Paola Merlo, Chundra Cathcart, Simon W. Townsend, Balthasar Bickel

**Affiliations:** ^1^ Department of Comparative Language Science, University of Zurich, Zurich, Switzerland; ^2^ Center for the Interdisciplinary Study of Language Evolution (ISLE), University of Zurich, Zurich, Switzerland; ^3^ Department of Evolutionary Anthropology, University of Zurich, Zurich, Switzerland; ^4^ Department of Linguistics, University of Geneva, Geneva, Switzerland; ^5^ Idiap Research Institute, Martigny, Switzerland

**Keywords:** whole-repertoire analysis, Markov chain models, call sequences, common marmosets, animal communication

## Abstract

Over the last two decades, an emerging body of research has demonstrated that non-human animals exhibit the ability to combine context-specific calls into larger sequences. These structures have frequently been compared with language’s syntax, whereby linguistic units are combined to form larger structures, and leveraged to argue that syntax might not be unique to language. Currently, however, the overwhelming majority of examples of call combinations are limited to simple sequences comprising just two calls which differ dramatically from the open-ended hierarchical structuring of the syntax found in language. We revisit this issue by taking a whole-repertoire approach to investigate combinatoriality in common marmosets (*Callithrix jacchus*). We use Markov chain models to quantify the vocal sequences produced by marmosets providing evidence for structures beyond the bigram, including three-call and even combinations of up to eight or nine calls. Our analyses of these longer vocal sequences are suggestive of potential further internal organization, including some amount of recombination, nestedness and non-adjacent dependencies. We argue that data-driven, whole-repertoire analyses are fundamental to uncovering the combinatorial complexity of non-human animals and will further facilitate meaningful comparisons with language’s combinatoriality.

## 1. Introduction

Language is a hyper-combinatorial communication system whereby sounds are organized into units that recombine into even larger, often structured, units [1–5]. For example, the sounds /t/, /iː/ and /m/ can be combined to form the word team (/ti:m/), or, recombined to form the word meat (/mi:t/). Moreover, when exchanging the /t/ sound with a /d/ and an /r/ one can form a new meaningful word dream (/dri:m/). Semantically meaningful units such as team or dream can be further combined to form larger phrases (i.e. the nominal phrase dream team), giving rise to the rich, hierarchical structures characteristic of language (e.g. the biologists and the linguists were a dream team for investigating the evolution of language).

Combinatoriality is also a key feature in non-human animal communication systems with research over the past five decades demonstrating that animals are capable of stringing sound units together to form structured and functionally holistic songs [6–10] but also context-specific, potentially meaningful calls, into larger meaning-bearing structures [11]. The latter have received considerable interest since they potentially correspond to at least some aspects of meaning composition in language [5,12], although it remains unresolved how far the similarities go and whether they are evolutionarily significant [13].

An important challenge is that, unlike human syntax, the overwhelming majority of studies on animal call combinations have identified sequences of just two calls, here termed bigrams [4–18]. For instance, southern pied babblers (*Turdoides bicolor*) and Japanese tits (*Parus minor*) combine alert calls with recruitment calls forming a bigram which prompts listeners to join the speaker and to mob potential predators [16,17,19]. Furthermore, Campbell’s monkeys (*Cercopithecus campbelli*) have been shown to affix an additional acoustic unit onto predator-specific alarm calls, leading to a potentially subtle meaning change regarding the preciseness of the threat [15], while the combination of two individually occurring alarm calls in the west African putty-nosed monkey (*Cercopithecus nictitans*) triggers a distinct behaviour, namely group movement [14].

One potential explanation for this apparent discontinuity between language and animal communication is that animals are constrained (whether at the production or the processing level) in their deployment of combinations above two call units [20,21]. Alternatively, it could be that researchers have generally failed to take a ‘whole-repertoire’ approach (although see [22]) whereby a considerably larger range of vocal output, from all calls in an animal’s known repertoire, is considered. Here, we apply such a quantitative approach to explore the combinatorial nature of common marmoset vocal strings with an emphasis on combinations beyond the bigram.

The common marmoset (*Callithrix jacchus*) is a small-bodied, arboreal American monkey species living in the dense tropical forests of Brazil. Common marmosets are highly social, residing in family groups where individuals cooperate to raise offspring [23,24].

Marmosets have also been shown to be highly vocal and extremely flexible in their vocal communication, with vocal input influencing call ontogeny in infants, and adults frequently engaging in turn-taking, whereby they variably adjust the intensity, timing and structure of their vocalizations [25–32]. Critically, previous work has demonstrated a tendency in marmosets for stringing calls together into longer sequences [28,33,34], though a precise quantification of the structures produced is missing.

Inspired by their successful application in gestural systems, animal song and language data, we applied Markov chain modelling to capture the sequential dynamics of marmoset combinatorial structures [10,35]. Specifically, we investigated whether marmosets produce sequences of calls that extend beyond bigrams and, if so, how these structures are organized.

## 2. Methods

### 2.1. Vocal recordings

Marmoset vocal data were collected through focal sampling [36] of eight individuals living in captivity in breeding pairs (one female and one male, see electronic supplementary material, table S1). A total of 21 h of audio recordings documented all occurrences of vocalizations from the focal individuals [33]. Focals were recorded 10 min at a time and on 16 different occasions. Each 10 min session was recorded in one of six controlled behavioural contexts (see electronic supplementary material, table S2). While all individuals were the focal for the same amount of time, which individual was the focal in a specific session and in which behavioural context this individual was recorded was determined randomly for each 10 min recording (see electronic supplementary material, §1).

To ensure there was no overlap of calls or false identification of the individual that was calling, the vocal output of the non-focal breeding partners was live-coded, recorded and annotated in addition, but excluded from the subsequent analysis.

### 2.2. Behavioural contexts

Previous research has suggested that physical distance between an individual and its conspecifics, plus the distribution of favoured food increases the frequency of call production in common marmosets [33,37]. In order to maximize the amount of data collected, we therefore recorded subjects in behavioural contexts that varied across two dimensions, namely physical distance between the monkeys and availability and distribution of food (see electronic supplementary material, table S2).

### 2.3. Data processing

**Figure 1.**
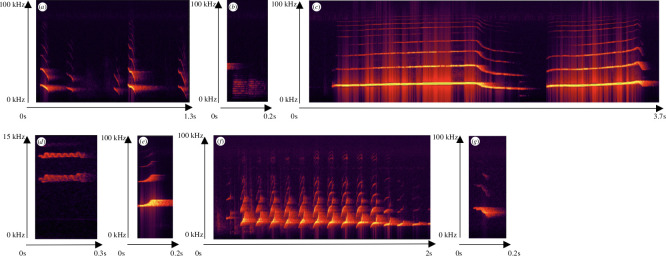
Spectrograms of the different call types making up the common marmoset vocal repertoire. (*a*) *chirp* call, (*b*) *ek* call, (*c*) *phee* call, (*d*) *trill* call, (*e*) *tsk* call, (*f*) *twitter* call and (*g*) *whistle* call.

**Figure 2.**
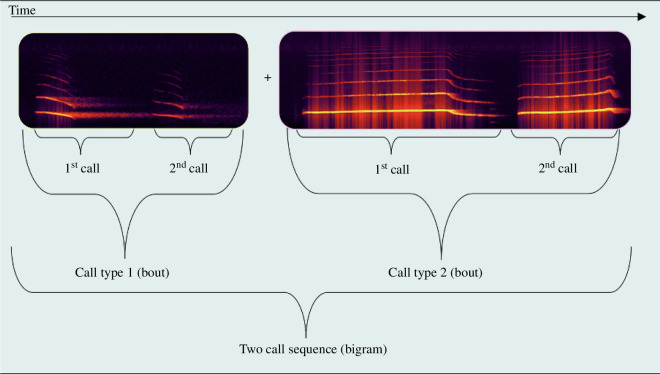
Description of how call sequences are built up. Call types are given in bouts consisting of one or more calls of the same type. Call sequences, in this example, a bigram, are then in turn composed of one or multiple call types that are uttered with silent pauses less than or equal to 0.5 s.

We manually annotated the recorded data in Avisoft SASLab Pro [38], noting the beginning and end of each uttered call type. Call types were given in bouts, meaning that they generally consisted of repeated instances of the same call not separated from each other by more than 0.5 s (see figures 1 and 2).

Call types were identified based on existing descriptions of common marmosets’ vocal repertoire [28,39–41], see electronic supplementary material, table S3. In line with this previous research, we defined eight broad call type categories. However, we excluded one call type, known in the literature as the *chatter*, from all subsequent analyses, since this call type only occurred once in the whole dataset.

We then read out possible call combinations, which were defined as sequences of call types that were uttered with a maximum of 0.5 s between one call type and the next [25,28,33] and figure 2.

To quantify the level of agreement between observers, we calculated Cohen’s kappa [42]. Accordingly, a trained observer independently coded 5% of the data over all focal individuals and all behavioural contexts in which the animals were recorded. We achieved a high level of agreement (kappa = 0.96), suggesting the annotation of call types was highly reliable.

### 2.4. Markov models

To explore the internal structure in common marmoset call sequences, we analysed them with Markov models, evaluating the transition probabilities in the data through a model’s ability to predict the next call in a sequence.

Specifically, in the first step, we modelled first-order (discrete-time) Markov chains. First-order Markov chains follow the Markov assumption that only the present state (in this case the present call type) matters when predicting the subsequent state (i.e. the following call type). In this model, a transition probability matrix represents the probability of moving from one state to the next state, determined by the conditional probability *p*(*s*_*i*_|*s*_*i*−1_), i.e. the probability of a state *s*_*i*_ given the preceding state *s*_*i*−1_. For this model, we included all seven call types as states in the model. In addition, we added a Start state and a Stop state, indicative of the start of a sequence or the end of a sequence, respectively. Adding a Start and a Stop state can inform if certain calls are more probable to start or end a call sequence.

We also modelled Markov chains over contextually broader states instead of call types, exploring the impact that contextual use could have on organizing marmoset call sequences. Based on previous research, we defined four states along the broad contextual categories of *Alarm* (for calls uttered in predatory situations), *Food* (for calls signalling the presence of food), *Contact* (for calls used to contact conspecifics and signal one’s own position) and *Mobbing* (for calls produced during inter- or intra-group aggression) (see [28,39–41], table 1 and electronic supplementary material, table S3). Delineating contexts can help shed light on the potential role of this on the sequential structure of vocalizations. To illustrate, it could be that contact calls always appear in the same position in a sequence, no matter whether the call in question is a close or a long-distance contact call (i.e., a *trill* or a *phee* call).

**Table 1.** Contextual categories and corresponding call types. For a closer description of contexts, see electronic supplementary material, table S3.

*Alarm*	*Food*	*Contact*	*Mobbing*
*whistle*	*chirp*	*phee, trill, twitter*	*ek*, *tsk*

Next, in order to examine longer sequences, we modelled second-order Markov chains, where the prediction of the subsequent state (i.e. call type or its contextual category) not only depends on the present state but also on the previous one.

To assess the extent to which the observed transition probabilities deviate from chance we also report the log-likelihood (loglik) of the observed transitions, i.e. the degree to which these would be expected if there was no structure and if each call type would appear with the probability estimated by its relative frequency in the data. The lower these log-likelihoods, the more the sequences deviate from simple chance co-occurrence. Log-likelihoods approach their maximum of 0 when the observed transition probability is expected already just because of the relative frequencies of the participating calls.

As an alternative, we use a Bayesian framework to infer values of transition probabilities most compatible with the data, assuming that these probabilities are drawn from the Dirichlet distribution (electronic supplementary material, §4).

This allows model comparison to assess the extent to which orders capture generalizations in our dataset by evaluating the predictive performance of different models on held-out call sequences. We use this to infer which model best explains our data and is thus the most probable. We use leave-one-out cross-validation, holding out a single sequence at a time and evaluating the log-likelihood of the sequence under a Markov model trained on the remaining sequences (see electronic supplementary material, §5). Since held-out sequences might not contain all states, we smooth them by a constant (*k*), chosen with variation to assess robustness [43].

The analyses were implemented using the statistical environment R (v. 4.1.1) and the R packages ‘tidyverse’ (v. 2.0.0), ‘dplyr’ (v. 1.1.4), ‘ggplot2’ (v. 3.5.1), ‘markovchains’ (v. 0.9.5), ‘slider’ (v. 0.3.1), ‘stringr’ (v. 1.5.1) and ‘viridis’ (v. 0.6.5), while the model comparison was carried out in Python (v. 3.12.4) using the packages ‘numpy’ (v. 1.17.2), ‘scipy’ (v. 1.3.1); graphics were generated with ‘matplotli’ (v. 3.1.1) [44–54].

## 3. Results

We recorded a total of 8497 calls from eight individuals. This included 4258 unigrams with the rest being organized into sequences of 645 bigrams, 770 trigrams, 87 quadrigrams and 41 sequences longer than a quadrigram, with the longest sequences being made of nine calls (see electronic supplementary material, table S4).

### 3.1. First-order Markov chains

**Figure 3.**
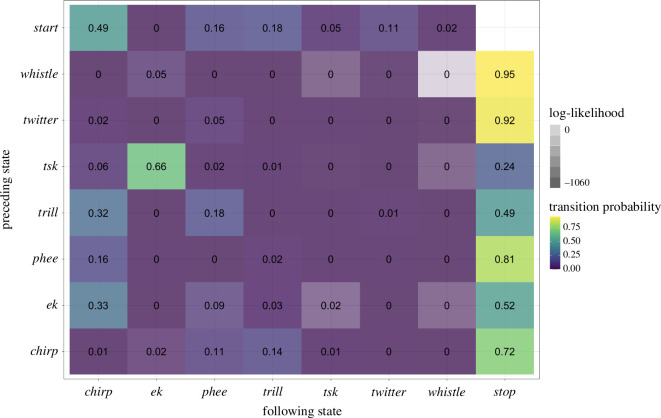
Adjacency matrix of the first-order Markov model depicting the transition probability from a call state (rows) to the next (column). The colour of each cell represents the corresponding transition probability (from yellow—a very high probability—over green to violet—a very low probability). The transparency of colour in each cell corresponds to the log-likelihood of the observed transitions, with lower log-likelihoods indicating higher deviation from chance expectations.

The Markov chain model suggests that the transition probabilities of the different call types throughout the input call sequences differ extensively from one another (see figure 3 and electronic supplementary material, figure S3 for an alternative visualization). For example, the mobbing call *tsk* is the only call type that has a larger probability of continuing to another state, namely *ek*, than continuing to the *Stop* state (tp(*tsk-ek*) = 0.66, tp(*tsk-Stop*) = 0.24, see electronic supplementary material, table S3). Moreover, the *tsk-ek* sequence is less likely to occur by chance than the *tsk-Stop* sequence (loglik(*tsk-ek*) = −620.8, loglik(*tsk-Stop*) = −15.4). *Whistle* calls also transition to *ek* but with much less probability and non-chance evidence. They are, in contrast to *tsk*, much more likely to instead transition to *Stop* (cf. tp(*whistle-ek*) = 0.05 versus tp(*whistle-Stop*) = 0.95; loglik(*whistle-ek*) = −3.3) versus loglik(*whistle-Stop*) = −27.5). In general, *ek* is not seen in the first position of a combination, i.e. does not follow immediately after the *Start* state (tp(*Start-ek*) = 0.001 and loglik(*Start-ek*) = −138.4).

According to the transition probabilities, the contact call types *trill* and *phee* seem to be core combinatorial units for common marmoset call sequences. For example*,* all but two call types transition to the *phee* state (tp(*Start-phee*) = 0.16, tp(*chirp-phee*) = 0.11, tp(*ek-phee*) = 0.09, tp(*trill-phee*) = 0.18, tp(*twitter-phee*) = 0.05). However, when considering the log-likelihood values, *Start-phee* and *trill-phee* are less likely to occur by chance than the others (loglik(*Start-phee*) = −65.2, loglik(*chirp-phee*) = −6.1, loglik(*ek-phee*) = −3.3, loglik(*trill-phee*) = −30.3, loglik(*twitter-phee*) = −18.3).

Finally, *chirp* calls have the highest probability of initiating and terminating a sequence, which could suggest that *chirp* calls, same as *whistle* calls, tend to occur alone (tp(*Start-chirp*) = 0.49, tp(*chirp-Stop*) = 0.72; loglik(*Start-chirp*) = −944, loglik(*chirp-Stop*) = −551.9). Furthermore, *chirp* calls are also commonly both preceded by *ek* and *trill* calls and regularly transition into *trill* calls (tp(*ek-chirp*) = 0.33, tp(*trill-chirp*) = 0.32, tp(*chirp-phee*) = 0.11, tp(*chirp-trill*) = 0.14; loglik(*ek-chirp*) = −5.4, loglik(*trill-chirp*) = −12.2, loglik(*chirp-trill*) = −14.9, see electronic supplementary material, figure S3.

**Figure 4.**
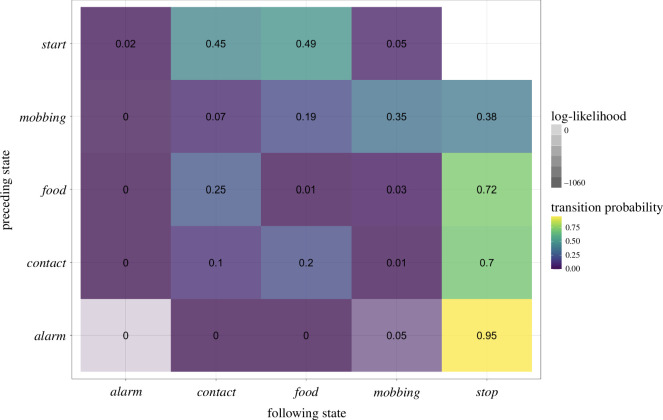
Adjacency matrix of the first-order Markov model depicting the transition probability of the four contextual categories *Alarm, Contact, Food and Mobbing*. The colour of each cell represents the corresponding transition probability (from yellow—a very high probability—over green to violet—a very low probability). The transparency of colour in each cell corresponds to the log-likelihood of the observed transitions with lower log-likelihoods indicating higher deviation from chance expectations.

Similarly to the call state model, the probability of transitioning from the contextual category *Alarm* to a state in a category other than *Stop* is very low, confirming that the *whistle* calls that make up the contextual category *Alarm* do not seem to play a central role in call combinations (tp(*Alarm-Stop*) = 0.95; loglik(*Alarm*-*Stop*) = −27.5; figure 4 and, for an alternative visualization, electronic supplementary material, figure S4). In contrast, the *Mobbing* category state, consisting of the mobbing calls *tsk* and *ek*, is the only state that shows almost the same probability to do a self-loop (i.e. transitioning to yet another call of the state *Mobbing*) as to transition to *Stop* (cf. tp(*Mobbing-Mobbing*) = 0.35 and tp(*Mobbing-Stop*) = 0.38). However, the log-likelihood scores of a call from the *Mobbing* state followed by another call from the *Mobbing* state deviates considerably more from chance than *Mobbing* to *Stop* or also *Mobbing* to any other call (i.e. loglik(*Mobbing*-*Alarm*) = −4.2, loglik(*Mobbing-Contact*) = −71.5, loglik(*Mobbing*-*Food*) = −10.4, loglik(*Mobbing-Mobbing*) = −303.5, loglik(*Mobbing-Stop*) = −4.2, see figure 4).

### 3.2. Second-order Markov chains

**Figure 5.**
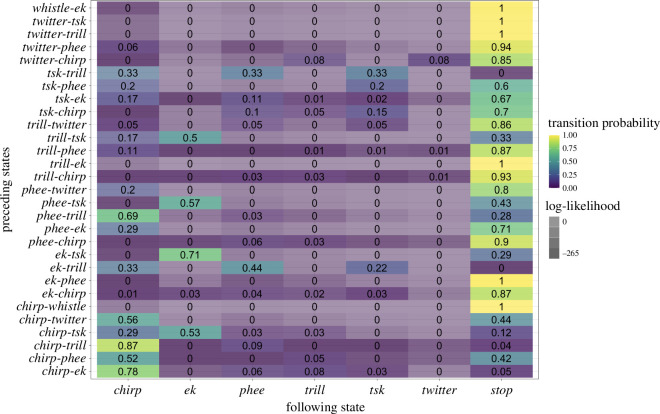
Adjacency matrix of the second-order Markov model depicting the probability of transitioning from a bigram (rows) to a third call (columns). The colour of each cell represents the corresponding transition probability (from yellow—a very high probability—over green to violet—a very low probability). The transparency of colour in each cell corresponds to the log-likelihood of the observed transitions with lower log-likelihood values indicating higher deviation from chance expectations.

The second-order Markov model assumes that the prediction of a state is dependent on two previous states, not just one (figure 5 and electronic supplementary material, §4 show virtually identical results from an alternative Bayesian analysis). For this first part, the states again represent the seven different call types comprising the repertoire (figure 1).

Particularly noteworthy is that the second-order Markov model matrix indicates that the probability for a chirp call being preceded by a bigram that is also initiated with a chirp call (i.e. *chirp*-x, ‘x’ being a representative for a variety of call types) is always relatively high and deviates from chance (tp([*chirp-ek*]-*chirp*) = 0.78, tp([*chirp-phee*]-*chirp*) = 0.52, tp([*chirp-trill*]-*chirp*) = 0.87, tp([*chirp-twitter*]-*chir*p) = 0.56; loglik([*chirp-ek*]-*chirp*) = −22, loglik([*chirp*-*phee*]-*chirp*) = −30.5, loglik([*chirp-trill*]-*chirp* = −244.8, loglik([*chirp-twitter*]-*chirp*) = −2.4; figure 5, lower left corner). One exception is the [*chirp-tsk*]-*chirp* sequence, which is less probable (tp = 0.29, loglik = −2.1; *chirp-tsk* is more likely to transition to *ek*; tp = 0.53, loglik = −54.5).

In addition, transitioning from a bigram with a *chirp* call in first position to the *Stop* state (i.e. the end of a sequence) is always less probable than transitioning to another *chirp* call (tp([*chirp-ek*]-*chirp*) = 0.78, tp([*chirp-ek*]-*Stop*) = 0.05, tp([*chirp-phee*]-*chirp*) = 0.52, tp([*chirp-phee*]-*Stop*) = 0.42, tp([*chirp-trill*]-*chirp*) = 0.87, tp([*chirp-trill*]-*Stop*) = 0.04, tp([*chirp-tsk*]-*chirp)* = 0.29, tp(*[chirp-tsk*]-*Stop*) = 0.12, tp([*chirp-twitter*]-*chirp*) = 0.56, tp([*chirp-twitter*]-*Stop*) = 0.44; loglik([*chirp-ek*]-*chirp*) = −22, loglik([*chirp-ek*]*-Stop*) = −33.7, loglik([*chirp-phee*]-*chirp*) = −30.5, loglik([*chirp-phee*]-*Stop*) = −16, loglik([*chirp-trill*]-*chirp* = −244.8, loglik([*chirp-trill*]-*Stop*) = −265.2, loglik([*chirp-tsk*]-*chirp*) = −2.1, loglik([*chirp-tsk*]-*Stop*) = −11.2, loglik([*chirp-twitter*]-*chirp*) = −2.4, loglik([*chirp-twitter*]-*Stop*) = −1.8; see figure 5).

Another striking pattern concerns calls with the mobbing call *tsk* in second position (‘*x-tsk*’). Except for *twitter-tsk*, *x-tsk* sequences have a higher probability to transition to *ek* than to *Stop* (tp([*ek-tsk*]*-ek*) = 0.71, tp([*ek-tsk*]*-Stop*) = 0.29, tp([*chirp-tsk*]*-ek*) = 0.53, tp([*chirp-tsk*]*-Stop*) = 0.12, tp([*phee-tsk*]*-ek*) = 0.57, tp([*phee-tsk*]*-Stop*) = 0.43, tp([*trill-tsk*]*-ek*) = 0.5, tp([*trill-tsk*]*-Stop*) = 0.33, tp([*twitter-tsk*]*-ek*) = 0, tp([*twitter-tsk*]*-Stop*) = 1; loglik([*ek-tsk*]*-ek*) = −16.7, loglik([*ek-tsk*]*-Stop*) = −2, loglik([*chirp-tsk*]*-ek*) = −54.5, loglik([*chirp-tsk*]*-Stop*) = −11.2, loglik([*phee-tsk*]*-ek*) = −12.6, loglik([*phee-tsk*]*-Stop*) = −1.7, loglik([*trill-tsk*]*-ek* = −9.3, loglik([*trill-tsk*]*-Stop* = −1.7, loglik([*twitter-tsk*]*-ek*) = −0.0, loglik([*twitter-tsk*]*-Stop*) = −1.6; see figure 5).

Furthermore, the bigram *tsk-ek* deviates from chance when transitioning to *chirp* or *phee* (tp([*tsk-ek*]-*chirp*) = 0.17, tp([*tsk-ek*]-*phee*) = 0.11; loglik([*tsk-ek*]-*chirp*) = −11.5, loglik([*tsk-ek*]-*phee*) = −11). However, *tsk-ek* sequences mostly end (tp([*tsk-ek*]-*Stop*) = 0.67; loglik([*tsk*-*ek*]*-Stop*) = −4.8).

**Figure 6.**
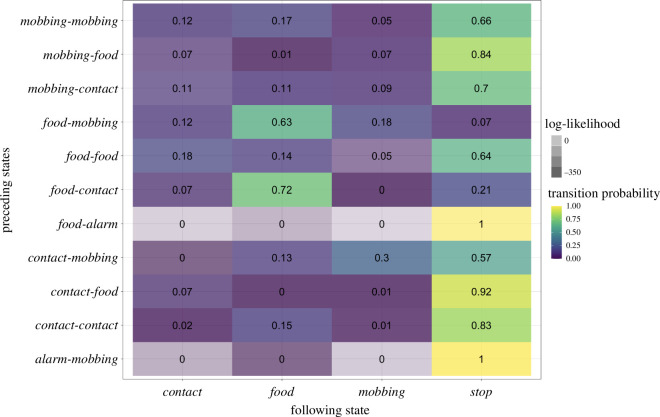
Adjacency matrix of the second-order Markov model depicting the probability transitioning from a bigram (rows) into a third call (column). The colour of each cell corresponds to the corresponding transition probability (from yellow—a very high probability—over green to violet—a very low probability). The transparency of colour in each cell corresponds to the log-likelihood of the observed transitions with lower log-likelihoods indicating higher deviation from chance expectations.

In a further step, we used the second-order Markov model to assess the interconnectivity and relevance of the three contextual categories *Contact*, *Food* and *Mobbing* (see figure 6). The category *Alarm* was not included in this as the call type *whistle*, which makes up the *Alarm* category, was not found in sequences longer than bigrams.

Similarly to the findings in the second-order Markov model, the category *Food*, followed by either the *Mobbing* or the *Contact* state (i.e. *Food-Contact*/*Mobbing*), is most likely to transition to the *Food* state again (tp([*Food-Contact*]-*Food*) = 0.72, tp([*Food-Mobbing*]-*Food*) = 0.63; loglik([*Food-Contact*]-*Food*) = −347.2, loglik([*Food-Mobbing*]-*Food*) = −17). In contrast, all bigrams ending in the *Food* category (i.e. x-*Food*, ‘x’ standing for either *Contact* or *Mobbing*) have a high probability to instead terminate the sequence with a transition to the *Stop* state (tp([*Contact-Food*]-*Stop*) = 0.92, tp([*Mobbing-Food*]-*Stop*) = 0.84; loglik([*Contact-Food*]-*Stop*) = −101.5, loglik([*Mobbing-Food*]-*Stop*) = −9.5).

Together with the results from the first-order Markov model, this indicates that, in general, the *Food* state has a high probability of transitioning to a *Stop*, but not when the *Food* state is followed by the *Mobbing* or *Contact* category. Under these conditions, an additional transition to the *Food* state is more likely.

Finally, we found a high transition probability between the bigram *Contact-Mobbing*, followed again by the category *Mobbing*. This result is also supported by log-likelihood values suggestive that [*Contact-Mobbing*]-*Mobbing* occurs more than expected by chance (tp([*Contact-Mobbing*]-*Mobbing* = 0.3; loglik([*Contact-Mobbing*]-*Mobbing*) = −13.7). Similarly, the log-likelihood value for *Mobbing-Mobbing* to *Contact* and *Food* suggests a deviation from chance as well (tp([*Mobbing-Mobbing*]-*Contact*) = 0.12, tp([*Mobbing-Mobbing*]-*Food*) = 0.17; loglik([*Mobbing-Mobbing*]-*Contact*) = −6.1, loglik([*Mobbing-Mobbing*]-*Food*) = −12.8).

### 3.3. Model comparison

**Figure 7.**
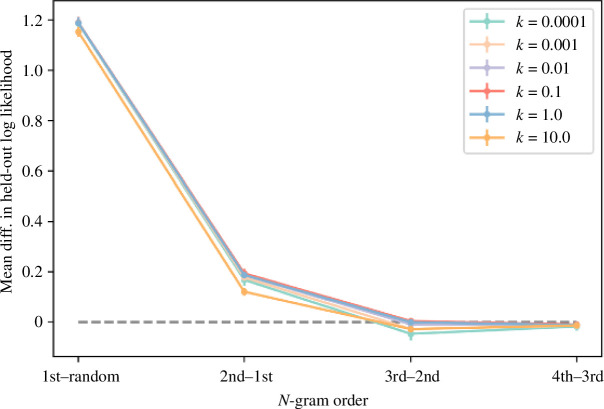
Pairwise differences (means and ±2 standard errors) in log-likelihoods under leave-one-out cross-validation between Markov models and their immediately simpler counterparts, for different additive smoothing constants k. See electronic supplementary material, figure S6 for the results of alternative models without the *Stop* state.

Above, we demonstrate that first- and second-order Markov models reveal recurrent statistical properties of marmoset call sequences. At the same time, however, these analyses do not address the question of which model best captures the structural properties of call sequences. In response, we investigated the pairwise log-likelihood differences under leave-one-out cross-validation between each Markov model and its immediately simpler counterpart (e.g. first-order versus random or chance, etc.). Figure 7 plots these pairwise differences along with ±2 standard errors across different values of the smoothing constant k. (An alternative set of models that exclude the *Stop* state yields very similar results; electronic supplementary material, figure S6). The differences between the second-order and first-order models include only positive values, which indicates that the second-order model is a better fit than the first-order model. In addition, the differences between the third-order and second-order models include mostly negative values, which indicates that the second-order model is a better fit than the third-order model as well. In sum, the majority of settings explored here suggest that the second-order Markov model appears to account for patterns in marmoset call combinations best out of all models surveyed.

## 4. Discussion

Through taking a whole-repertoire approach, we show that common marmosets systematically string calls together into larger, potentially internally structured sequences. Specifically, we were able to show that marmosets create sequences of call types that extend beyond the bigram, including structures comprising up to nine calls. Particularly noteworthy is that, contrary to the prevailing consensus in the field, bigrams are not the limit of combinatorial structuring in common marmosets. In fact, larger trigram structures seem to be more prevalent in the dataset than bigrams (see electronic supplementary material, table S4).

**Figure 8.**
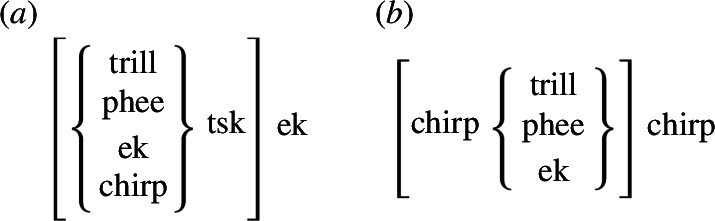
The hierarchical structure suggested by the second-order Markov model. Elements in curly brackets are options to choose from, while the square brackets define constituents between which the second-order transition probabilities hold.

In addition to uncovering structures that go beyond the bigram length, a pertinent additional finding from the Markovian analysis is the potential internal structuring of call sequences (see figure 8). A trigram, generally, consists of three calls, yet precisely how these three calls are structured can take various forms. It could be, for example, that calls are ordered according to adjacency, namely that one only predicts the next (i.e. ${call}_{1}$ predicts ${call}_{2}$ and ${call}_{2}$ then predicts ${call}_{3}$). However, our results suggest that this kind of first-order adjacency is insufficient to explain our data. In fact, the best-fitting model requires a second-order Markov model, where the probability of ${call}_{3}$ depends not on ${call}_{2}$ alone but on the combination of ${call}_{1}$ and ${call}_{2}$. For example, the transition probability from *tsk-ek* to *phee* cannot be reduced to the product of the transition probability from *tsk* to *ek* and the transition probability from *ek* to *phee* (see figures 3 and 5 and electronic supplementary material, figure S3).

The transition probabilities we see here are therefore more likely to arise from an underlying structure, not simple adjacency chains. Two variants of this structure emerge, both involving hierarchical nesting and some amount of recombination where more than one call participates in the structure (symbolized as ‘*x*’ here, ranging over more call types). The first structure is [*x-tsk*]-*ek*, with *x* ranging over *trill*, *phee*, *ek* and *chirp*. Potentially similar is the [*tsk*-ek]-*x* structure but here *x* is most likely *Stop*, with *chirp* and *phee* having lower probabilities. Importantly, the *tsk-ek* sequence is not the same in these two cases because the second-order transition probability between [*x-tsk*] and *ek* cannot be reduced to that between *x* and *tsk* and between *tsk* and *ek*; similarly, the second-order transition probability between [*tsk-ek*] and *x* cannot be reduced to that between *tsk* and *ek* and between *ek* and *x*. The corresponding first-order Markov model fits the data considerably less well (figure 7). In light of this, the high transition probability from *tsk* to *ek* in the first-order model seems inflated because it arises from two different structures, [*x-tsk*]-*ek* and [*tsk-ek*]-*x*.

The second structure is [*chirp-x*]-*chirp*, where *x* ranges over *ek*, *phee*, *trill* and, to a lesser extent, *twitter*. In fact, when reviewing the sequences found most frequently in the datasets, not only were the three most common trigrams found to start and end with a *chirp* call, but the same was true for the three most common quadrigrams (see electronic supplementary material, table S5). However, given the fact the second-order model fits the data better, it is unlikely that *chirp* simply serves to initiate and end a call sequence. Again, the structure cannot be reduced to a transition between *chirp* and *x* and between *x* and *chirp*.

Both structures involve non-adjacent dependencies, at least between calls of the same type: *ek* and *ek* (figure 8*a*) and *chirp* and *chirp* (figure 8*b*) are related to each other (they are the same) but they are non-adjacent not only in the linear string, but, critically, in hierarchical structure. Our findings here represent, to our knowledge, some of the first evidence of the presence of such structures in the production of a natural call-based non-human animal communication system. This is consistent with the finding that common marmosets have a latent capacity to parse nested non-adjacent structures [55,56].

What remains unresolved, however, is how these structures are generated. Most pressingly, we do not know whether there are (like in language) specific relationships of meaning involved, so that *tsk-ek* means something different in the [*x-tsk*]*-ek* and the [*tsk-ek*]*-x* structure (as would be the case in language, where the meaning of an expression like ‘Swiss history teacher’ depends on whether its bracketed as ‘[Swiss history] teacher’ or as ‘Swiss [history teacher]). Another limitation of our findings is that we have confined ourselves to Markov models and have not yet assessed whether more complex models would predict the data even better.

Similar findings of internal structure have recently been forwarded in the chimpanzee communication system, namely where bigrams are recombined to be either preceded or followed by an additional single call [22]. The presence of such abilities in primate species both closely and distantly related to humans could be suggestive of a common evolutionary origin in the last common ancestor of humans and monkeys, approximately 45 million years ago [57,58]. However, it could also be that the unique highly cooperative social and breeding system that marmosets share with humans has been a major driver in selecting for communicative complexity, one dimension of which is combinatoriality ([24], though see [26,34,40,59–64] for more examples regarding other dimensions of complexity in the common marmoset’s communication system). Further work on other great ape and monkey vocal, but also gestural or multimodal, systems as well as studies on non-primate species with similar cooperative breeding systems, is key to disentangling these alternatives.

In this article, we applied Markov models to not only call types, but also broader contextually defined call categories in order to investigate the potential influence of behavioural context on the combinatorial abilities of the common marmoset. This categorization relies on the assumption that it is clear to what categories the different call types belong and that no ambiguity in call meaning exists. While this might be the case for some calls, for others such a characterization may be premature. For example, *twitter* calls have been classified as social contact calls by many [39,65–67], yet, *twitters* might also encode an additional level of alertness not present in other contact calls, namely, *trills* as close-range contact calls and phees as long-distance contact calls. This suggests that *trills* and phees are functionally and contextually more similar than either are to *twitters*. In the future, more detailed contextually based analyses will help shed further light on the degree and extent of context-specificity of marmoset vocalizations and whether certain vocalizations are more ambiguous in their information content.

Finally, since our findings derive from a captive population of marmosets, follow-up work could compare the results presented here with wild-living common marmosets to ascertain the extent to which the structures detected are specific to, or a result of, the captive environment.

In conclusion, by taking a whole repertoire approach and by employing Markov models, we show that common marmosets can string together context-specific calls, with the vast majority even extending beyond the bigram. Some of the identified combinations also seem to be internally structured, suggesting that marmosets adhere to certain constructional rules when stringing calls together. Additional contextual analyses and playback experiments are central to confirming the presence of such rules. Furthermore, while our approach allows differences in transition probabilities to be established, it cannot distinguish between the many ways in which such differences can be generated. More research is needed to disentangle the generative processes and underlying structures that lead to the patterns we found, possibly leveraging results from the analysis of animal song [8–10]. However, minimally, these data indicate that internally structured call combinations in animals, extending beyond simple bigrams, might be more common than previously thought.

## Data Availability

The data and the corresponding scripts used for analysis can be found on the Open Science Framework (OSF) [68]. Supplementary material is available online [69].
